# Antifungal Activity of Beluntas “Indian Camphorweed” ( *Pluchea indica* ) Ethanol Extract on *Candida albicans In Vitro* Using Different Solvent Concentrations

**DOI:** 10.1055/s-0041-1736591

**Published:** 2021-12-22

**Authors:** Wayan Larissa Demolsky, Vinna Kurniawati Sugiaman, Natallia Pranata

**Affiliations:** 1 Department of Oral Biology, Faculty of Dentistry, Maranatha Christian University, Bandung, West Java, Indonesia

**Keywords:** *Candida albicans*, nystatin, amphotericin B, Indian camphorweed, *Pluchea indica*, beluntas

## Abstract

**Objective** Oral candidiasis is an infection caused by pathogenic fungi *Candida albicans* , with a considerably high prevalence of 20 to 72%. Indian camphorweed ( *Pluchea indica* ) also known as “beluntas” as the local name has been known as a traditional medicine in Indonesia. The objective of this study is to research the minimum inhibitory concentration (MIC) and the minimum fungicidal concentration (MFC) of beluntas ethanolic extract against the growth of *C. albicans* .

**Materials and Methods** The MIC and MFC were measured by microdilution assay and total plate count respectively with a variation of solvents (DMSO 1%, 10%, and 4%) and beluntas extract with concentrations between 0.3125 and 200 mg/mL. Amphotericin and nystatin were used as a comparison.

**Statistical Analysis** One-way *analysis of variance* and posthoc Tukey test were used to determine the significant difference between treatments.

**Results** It was found that the MIC ranged from 50 to 200 mg/mL in the test with DMSO 10% solvent and MFC was found to be at a concentration of 200 mg/mL. However, there is a significant inhibitory effect and killing effect from DMSO 10% against *C. albicans* ( *p* = 0.000). MIC was also found within concentrations of 100 mg/mL of beluntas extract in DMSO 4%. In this study, the DMSO 4% concentration neither showed significant inhibitory effects nor killing effects; therefore, the result was acceptable ( *p* = 0.357).

**Conclusion** Ethanol extract of beluntas ( *P. Indica* ) has the potential of being an antifungal agent with inhibitory activity in concentrations ≥100 mg/mL, which is similar to nystatin ( *p* = 0.278). The MFC for the extract was above 100 mg/mL, which cannot be measured with this method as a higher concentration of DMSO is needed, which had a toxic effect on the tested fungi.

## Introduction

*Candida albicans* and other *Candida* species colonized up to 75% in a healthy person's oral cavity. *Candida albicans* is an opportunistic pathogen which can cause a wide range of disease manifestation from mild oral disease to disseminated candidiasis. This event can be triggered by several conditions including immunosuppression, endocrine imbalance, prolonged antibiotic therapy, smoking, and chemotherapy. 1 2

Nystatin and amphotericin from the polyene class are the first-line therapy for candidiasis which work by affecting the fungi membrane permeability and thereby causing cell death. However, several side effects had been reported regarding polyene formulation including delayed hypersensitivity attributed to cinnamic aldehyde and increase risk of carries due to sugar in the oral suspension. Rare cross-reactivity between nystatin and other macrolides and resistance to polyene antifungals have also been reported. 3 The most common adverse effects were poor taste and gastrointestinal adverse reactions. 4 Moreover, although rare, the resistance of fungi to polyene antifungal had been reported. 5 Thereby, novel drugs that are safer and less resistance inducer, especially coming from natural resources, have become the wide interest of research.

Indian camphorweed ( *Pluchea indica* ) also known as “beluntas” in the local name is a native plant of Indonesia that has been used as a traditional medicine. The plant's leaf has a unique aroma and bitter taste. This part is used as a gastrointestinal agent, a diuretic, and an antipyretic traditionally. It also has antiseptic properties and is used as a deodorant and vaginal leukorrhea medicine, which showed its potential as an antifungal agent. 6 7 This research is purposed to find the antifungal activity of Indian camphorweed ( *P. indica* )/beluntas against *C. albicans in vitro* as a novel natural-resource-based therapeutic for oral candidiasis.

## Materials and Methods

### Beluntas Leaf Ethanol Extract

Beluntas leaves were obtained from Balai Penelitian Tanaman Rempah dan Obat (BALITTRO) ( *Spices and Medicinal Plants Research Center* ), Bogor, West Java, Indonesia. Leaf extracts were obtained using a maceration method. Maceration is used for the extraction as this method is the simplest method of extraction, also can be used to extract polar and nonpolar fraction of the active compound and the thermolabile active compounds. 8 First, the leaves were dried using a food dehydrator and ground with a food processor. The leaf powder was later soaked in ethanol 70% for 3 days. The active compound will be dissolved in the solvent during soaking. Ethanol 70% was used as a solvent as this solvent is able to extract polar and nonpolar active compounds of the leaves and also is less cytotoxic. After 24 hours, the filtrate was taken and filtered with a filter paper. This process is necessary to eliminate leaf grounds which do not contain any active compounds anymore. Later the extract was concentrated using rotary evaporator. 9 For experiment, beluntas ethanol extract was diluted in DMSO 1%, 4%, and 10% with concentrations ranging from 10 to 0.3125 mg/mL, 100 to 3.125 mg/mL, and 200 to 3.125 mg/mL, respectively. DMSO 1% was used in the initial experiment by referring to the Clinical Laboratory Standard Institute (CLSI) M-27 protocol of standard solvent used for microdilution assay. 10 However, at this concentration we cannot find the minimum inhibitory concentration (MIC) and minimum bactericidal concentration (MBC) value as the maximum diluted extract is 20 mg/mL (see the Results section), hence we increase the DMSO concentration. Amphotericin and nystatin 0.25 mg/mL in 1%, 4%, and 10% DMSO was used as comparison.

### Preparation of *C. Albicans* Inoculum

*Candida albicans* ATCC 10231 was used for this study and prepared in accordance with the CLSI M-27 protocol. 10 Fungi were subcultured in Potato Dextrose Agar (Himedia M096) for 24 hours before used. The 24-hour old culture was used in the experiment as in this stage the yeast is in the log phase (actively budding). 11 Later approximately 1 loop of *C. albicans* was taken and dissolved in phosphate-buffered saline (Sigma Aldrich 11666789001). The turbidity of the suspension was adjusted to 0.5 McFarland turbidity standard (equals to 10 ^6^ CFU/mL of yeast), which is defined as 0.08 to 0.1 absorbance value measured in 600 nm wavelength, and further three times 10-fold diluted in Potato Dextrose Broth (PDB) (Himedia, M403) so the final inoculum concentration was approximately 1 × 10 ^3^ CFU/mL.

### Minimum Inhibitory Concentration

The 96-well plate microdilution assay was used for this purpose according to the CLSI M-27 protocol of antifungal susceptibility of yeast with slight modifications. 10 A 100 μL of inoculum suspension was loaded unto the wells, except for the blank wells and positive-control well. As much as 100 µL PDB was added to the blank wells and positive-control well. Thereby the positive-control well consists of *C. albicans* suspension and 100 µL medium without treatment. Later 100 µL of diluted extract and nystatin and amphotericin in DMSO was added to each well, including the blank wells. A well filled with PDB only was used as the negative control. To measure the inhibitory effect of the solvent, a “solvent control” well was added which consists of 100 µL *C. albicans* suspension and 100 µL DMSO 1%, 4%, and 10%. Each treatment was plated three times. The plate was later incubated at 37°C in an incubator for 24 hours, and later the absorbance was read at 530 nm wavelength. Until now, there has been no precise definition for MIC measured with absorbance. In its original definition, according to CLSI, the MIC value is the lowest concentration where there is no visual growth of microorganisms. 11 In one experiment, the MIC value was defined as the lowest concentration where there is a sharp decline in absorbance value. 12 In this experiment, MIC was defined as the lowest concentration which gives 95 to 100% inhibition. Growth inhibitory activity was calculated as follow:



### Minimum Fungicidal Concentration

Minimum fungicidal concentration (MFC) was determined through total plate count assay according to CLSI (2008) standard. 10 A 100 μL of suspension from wells with 100% inhibition, including the medium-only well itself, *C. albicans* untreated, and *C. albicans* in DMSO solvent, was platted to Potato Dextrose Agar and incubated for 24 hours. Serial dilution was performed if necessary to make colony counting easier. Each treatment was platted three times. The colony was counted and converted to *colony-forming unit* per mL, and %killing activity was calculated as follows:



MFC is the lowest concentration where there is 99.9% killing activity according to CLSI (2008) definition. 10

## Results

### Minimum Inhibitory Concentration

**Fig. 1:**
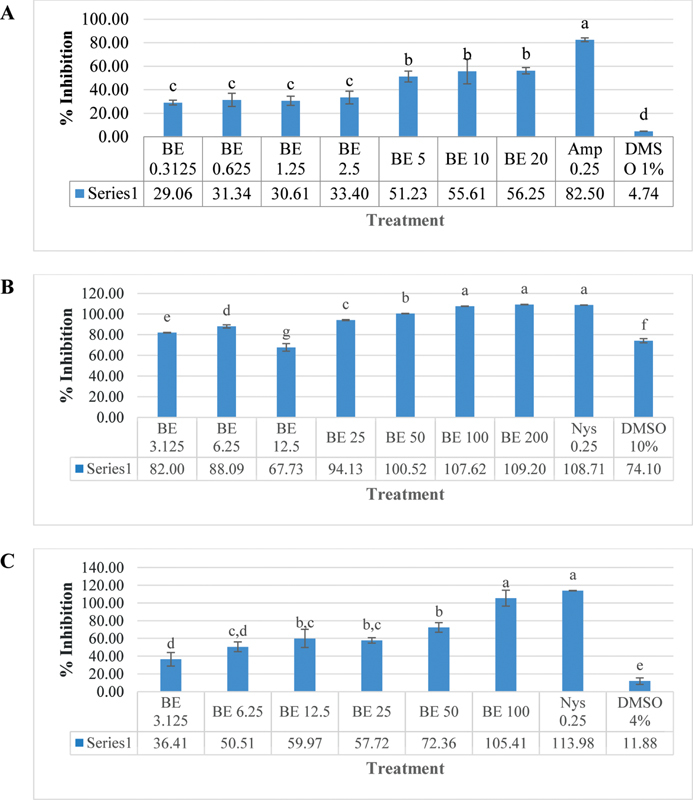
Percent of *C. albicans* growth inhibition after beluntas extract (BE) challenge in microdilution assay. Different solvents were used: ( **A** ) 1% DMSO, ( **B** ) 10% DMSO, ( **C** ) 4% DMSO which resulted in different maximum diluted extracts and different inhibitory activities. Different alphabets located on top of the bar show statistical difference between groups ( *p* > 0.05) as calculated with ANOVA and posthoc Tukey test. ANOVA, analysis of variance.

Different concentrations of solvent are used for this experiment. The first experiment used 1% DMSO as suggested by CLSI guidelines. 10 The maximum concentration of beluntas extract (BE) that can be dissolved in this solvent is 20 mg/mL. Amphotericin B was used as a comparison control for this setting. The maximum inhibitory activity that can be achieved by 20 mg/mL BE is 56.25%. The inhibitory activity is very low in this concentration. Amphotericin 0.25 mg/mL only inhibits 82.5% of *C .albicans* growth, where it is expected to give 100% inhibition. DMSO 1% did not have a toxic effect as it only inhibits 4.74% growth ( Fig. 1A ).

As the first set did not give MIC value, a second experiment was conducted where 200 mg/mL BE was used as the highest concentration diluted in DMSO 10%. The inhibitory activity was very high for this setting, ranging from 82% in 3.125 mg/mL to 100% in 50 to 200 mg/mL concentration. The control was changed to nystatin 0.25 mg/mL, which gave 100% inhibition. The inhibitory activity of BE 100 mg/mL and BE 200 mg/mL gave equal results with nystatin ( *p* = 0.960), thereby it will be plated for MFC analysis. However, DMSO 10% itself showed high inhibitory activity (74.1%) ( Fig. 1B ). This result may lead to interpretation bias of whether the inhibitory activity is the result of the DMSO or the extract.

As there was confounding effect from the inhibitory activity of DMSO, we conducted the next experiment using a lower DMSO concentration (4% DMSO). The maximum extract concentration that can be soluble is 100 mg/mL. Nystatin was still used as a comparison for this setting. BE 100 mg/mL showed 100% inhibition, comparable with nystatin ( *p* = 0.995). DMSO 4% only gave slight inhibitory activity of 11.88% ( Fig. 1C ).

### Minimum Fungicidal Concentration

**Fig. 2:**
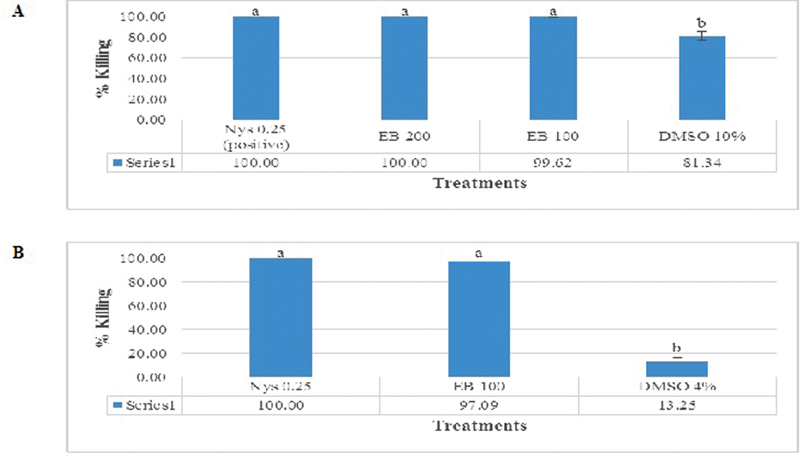
Percent of *C. albicans* killing as assessed with total plate count in ( **A** ) 10% DMSO and ( **B** ) 4% DMSO setting. Different alphabets located on top of the bar show statistical difference between groups ( *p* > 0.05) as calculated with ANOVA and posthoc Tukey test. ANOVA, analysis of variance.

Nystatin 0.25 mg/mL in DMSO 10% and BE 200 mg/mL in DMSO 10% showed fungicidal activity. Meanwhile, BE100mg/mL in DMSO 10% and DMSO4% only showed fungistatic activity (killing activity <99.9%). DMSO 10% showed a quite high killing activity (81.34%). However, DMSO 4% showed slight killing activity (13.25%). It can be safely concluded that DMSO 4% is a suitable concentration to carry out MIC and MFC experiments, which did not give significant inhibitory and killing activity ( Fig. 2 ).

## Discussion

This study showed beluntas ethanol extract activity toward growth inhibition of *C. albicans.* In the first setting, 20 mg/mL BE is not sufficient to induce a fungistatic effect; however, it still showed an inhibitory effect. This effect is still can be seen in the DMSO 10% setting where the inhibitory effect of each concentration is significantly higher in contrast with DMSO 10% alone. From the series of experiments, the MIC value of BE is 100 mg/mL, which did not differ from nystatin positive control. Our result is in line with previous research by Samalo, where the MIC of beluntas ethanol extract was at 16% concentration, while the MFC was at 20% using the macrodilution method. However, in this experiment, we cannot ascertain the type of solvent used and also the exact mg/mL concentration of the extract, as it is not fully accessible. 13 To our knowledge, this is the first research on beluntas ethanol extract activity toward *C. albicans* inhibition using microdilution assay with different concentrations of solvents used.

The antifungal activity of the ethanol extract of beluntas leaves is thought to be due to the synergistic effect of each secondary metabolite contained in the ethanol extract of beluntas leaves. Beluntas leaves contain flavonoids, phenols, saponins, tannins, steroids/triterpenoids, terpenoids, and alkaloids as reported in our previous study. 14 Based on the research of Widyawati et al, the ethanol extract of beluntas has levels of total flavonoids equivalent to 18,555 ± 1,792 mg CE/100 g dry weight and total phenolic equivalent to 16,958 ± 897 mg GAE/100 g dry weight. 15 In line with these research, here we found flavonoid content of 19.44 mg/g in dry beluntas leaves. 16

Flavonoids are known to be able to inhibit fungal growth through several mechanisms, namely, efflux pump inhibition, cell division inhibition, inhibition of RNA/DNA synthesis or fungal protein, inhibition of fungal cell wall formation, mitochondrial dysfunction, and disruption of the fungal plasma membrane. 17

Ergosterol is an important component in the formation of cell membranes. Phenols can inhibit ergosterol biosynthesis, and disrupt the cell membrane, which causes leakage of intracellular components and causes changes in the permeability of the fungal membrane. Deformation of the cell wall causes a significant reduction in cell size. Besides, phenols can also interfere with cell metabolism by inhibiting cell transports resulting in inhibition of fungal cell growth which resulted in apoptosis. 18 In more detail, phenol inhibits CYP51 enzyme activities and fungal squalene epoxidase, the first enzymes involve in the ergosterol biosynthesis pathway. 19

Saponin significantly induced the production of H _2_ O _2_ and resulted in membrane lipid peroxidation, thus leading to an increase in cell membrane permeability and the leakage of K(+), soluble protein, and soluble sugar. 20 Steroidal saponins are known to increase mitochondrial membrane potential, thus causing mitochondrial and reticulum endoplasm stress which leads to the internal apoptotic pathway. 21 22 Meanwhile, triterpenoid saponin induced accumulation of intracellular reactive oxygen species, resulting in mitochondrial dysfunction. It also breaks down the membrane barrier of *C. albicans* causing leakage of intracellular trehalose, entrance of extracellular impermeable substance, and decrease of ergosterol content. 23

In this experiment, two approved drugs from the polyene class were used as comparison control, nystatin and amphotericin B. Nystatin affects *C. albicans* by inhibiting the stages of glucose metabolism and influencing cell permeability, as a result, *Candida* cells will lack energy so they experience atrophy and over time their growth and multiplication are inhibited. 14 Amphotericin B works by binding to ergosterol which is the main component of fungal cell membranes which cause depolarization of fungal cells thus causing the fungal cells to die. 24 In this experiment, 0.25 mg/mL amphotericin did not give fungistatic effects. According to a previous study, the MIC of amphotericin B in *C. albicans* ATCC10231 was 0.25 mg/mL. 25 Probably amphotericin that was used in the experiment had degraded.

The MFC value cannot be found in this experiment because a higher concentration of beluntas is needed, while it can be met using DMSO 4%. A higher DMSO concentration will result in toxic effects to *C. albicans* , which may affect the interpretation of the result. We found that DMSO 10% is toxic. This result is in line with a previous research from Randhawa where DMSO 10% gave significant inhibitory effects toward *C. albicans* as DMSO can dissolve the fungal membrane, thereby a higher concentration will result in cell death. 26 Meanwhile, DMSO 4% is considered safe and can be used for experiments. 27 Lower concentration of DMSO 4% cannot disolve beluntas ethanol extract regarding to the hydrophobic nature of the extract, which will precipitate in lower concentration of DMSO. DMSO is a nonpolar solvent appropriate to dissolve various nonpolar compounds. 28

Although our study shows potential antifungal activity of beluntas ( *P. indica* ) leaves, several limitations had been met during the experiment. First, the only *C. albicans* strain used in the experiment is ATCC 10231. Second, a higher concentration of the extract is needed to carry out fungicidal activity analysis. While a higher concentration of DMSO is needed, to dissolve the extract (as the extract is not solvable in water) might be one issue, and resulted in DMSO cytotoxicity; the high concentration of extract itself may have a cytotoxic effect. Our previous study showed that ethanol extract of beluntas leaves has an IC _50_ value at 311.77 μg/mL against 3T3/Balb-C mice fibroblast cells. 16

Further research of BE activity against various *C. albicans* strains is needed to confirm its antifungal property. High performance liquid chromatography study might also be done to characterize the active compounds contained in the leaves and *in silico* study might be performed to find optimum solvents for the extraction. Another kind of assay or solvent is needed to find the exact value of BE MFC.

## Conclusion

From this study, we found that beluntas ( *P. indica* ) ethanol extract had inhibitory effects on *C. albicans* *in vitro* . The MIC value is 100 mg/mL. MFC value cannot be determined exactly because a higher concentration of DMSO is needed to dissolve the extract, which may have toxic effects on fungi.
